# Clinical Pathological Features and Current Animal Models of Type 3 Macular Neovascularization

**DOI:** 10.3389/fnins.2021.734860

**Published:** 2021-08-26

**Authors:** Wei Qiang, Ran Wei, Yongjiang Chen, Danian Chen

**Affiliations:** ^1^Research Laboratory of Ophthalmology and Vision Sciences, State Key Laboratory of Biotherapy, West China Hospital, Sichuan University, Chengdu, China; ^2^Department of Ophthalmology, West China Hospital, Sichuan University, Chengdu, China; ^3^The School of Optometry and Vision Science, University of Waterloo, Waterloo, ON, Canada

**Keywords:** age-related macular degeneration (AMD), animal model, Type 3 macular neovascularization, retinal angiomatous proliferation (RAP), multimodal imaging, von Hippel – Lindau, hypoxia inducible factor (HIF), retinoblastoma gene (Rb1)

## Abstract

Type 3 macular neovascularization (MNV3), or retinal angiomatous proliferation (RAP), is a distinct type of neovascular age-related macular degeneration (AMD), which is a leading cause of vision loss in older persons. During the past decade, systematic investigation into the clinical, multimodal imaging, and histopathological features and therapeutic outcomes has provided important new insight into this disease. These studies favor the retinal origin of MNV3 and suggest the involvement of retinal hypoxia, inflammation, von Hippel–Lindau (VHL)–hypoxia-inducible factor (HIF)–vascular endothelial growth factor (VEGF) pathway, and multiple cell types in the development and progression of MNV3. Several mouse models, including the recently built *Rb/p107/Vhl* triple knockout mouse model by our group, have induced many of the histological features of MNV3 and provided much insight into the underlying pathological mechanisms. These models have revealed the roles of retinal hypoxia, inflammation, lipid metabolism, VHL/HIF pathway, and retinoblastoma tumor suppressor (Rb)–E2F cell cycle pathway in the development of MNV3. This article will summarize the clinical, multimodal imaging, and pathological features of MNV3 and the diversity of animal models that exist for MNV3, as well as their strengths and limitations.

## Introduction

Type 3 macular neovascularization (MNV3) is a unique form of late-stage age-related macular degeneration (AMD), which has been discovered three decades ago, but its pathogenic mechanism is still elusive.

### Age-Related Macular Degeneration

Age-related macular degeneration is the leading cause of legal blindness in aged individuals in developed countries (Klein et al., 2002; Mitchell et al., 2018; Fleckenstein et al., 2021). AMD is a multifactorial disease related to aging, genetic susceptibility, and environmental risk factors. AMD affects photoreceptors, retinal pigment epithelium (RPE), Bruch membrane, and the choriocapillaris around the macular area (Fleckenstein et al., 2021). A systematic review showed that ∼8.7% of the worldwide population has AMD; there are nearly 196 million AMD patients all over the world in 2020, and this number will increase to ∼288 million in 2040 (Wong et al., 2014). According to the characteristics of the disease, AMD can be divided into two types: dry AMD and wet AMD (Ferris et al., 2013). Dry AMD accounts for about 85%, manifested as degeneration of RPE cells and photoreceptors; in severe cases, geographic atrophy (GA) forms. Wet AMD, also known as neovascular AMD (nAMD), accounts for about 15%, manifested as macular neovascularization (MNV). More than 80% of patients blinded by AMD are due to wet AMD (Miller, 2013). Clinically, AMD can be classified as early-stage (medium-sized and large drusen, pseudodrusen, and/or retinal pigment anomalies) and late-stage (nAMD and GA) (Klein et al., 2014; Mitchell et al., 2018).

Traditionally, nAMD is considered choroidal neovascularization (CNV) and is divided into occult (type 1) and classical (type 2) CNV (Macular-Group., 1991). Type 1 CNV refers to neovascular vessels confined to the sub-RPE space, and Type 2 refers to vessels proliferating above the RPE in the subretinal space (Gass, 1997). Recently, nAMD is renamed as MNV and classified into type 1 MNV, type 2 MNV, and type 3 MNV; polypoidal choroidal vasculopathy (PCV) is considered a subtype of type 1 MNV (also called aneurysmal type 1 neovascularization) (Spaide et al., 2020). Type 1 MNV is the occult (type 1) CNV, type 2 MNV is the classical (type 2) CNV, and type 3 MNV is retinal angiomatous proliferation (RAP) (Spaide et al., 2020).

### Type 3 Macular Neovascularization

**FIGURE 1:**
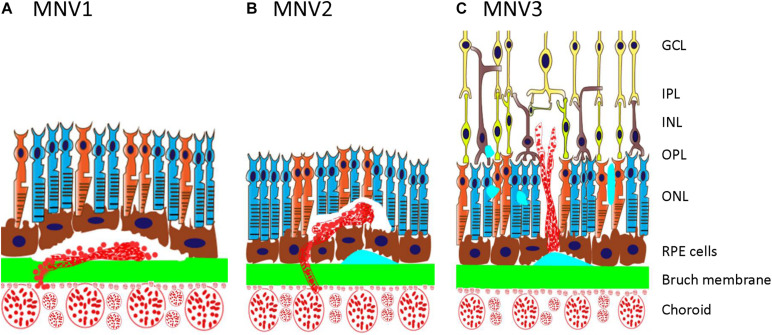
Schematic showing type 1, type 2, and type 3 MNV. **(A)** Type 1 MNV is an ingrowth of vessels originating from the choriocapillaris into the sub-RPE space. **(B)** Type 2 MNV is the proliferation of new vessels arising from the choroid into the subretinal space. **(C)** Type 3 MNV is a downgrowth of vessels from the retinal vascular plexus toward the outer retina. GCL, ganglion cell layer; IPL, inner plexiform layer; INL, inner nuclear layer; OPL, outer plexiform layer; ONL, outer nuclear layer; RPE, retinal pigment epithelium; MNV, macular neovascularization.

Type 3 macular neovascularization is an important subtype of nAMD (Figure 1), different from the type 1 or type 2 MNV as mentioned earlier (Yannuzzi et al., 2001; Freund et al., 2008). It was first reported in 1992, in which unusual RPE detachments were associated with retinal vessels that dove down into the deep retina and formed an angiomatous lesion (Hartnett et al., 1992). Kuhn et al. (1995) also described similar lesions by fluorescein and indocyanine green angiography and termed such lesions as chorioretinal anastomosis. Subsequently, it has also been referred to as deep retinal vascular anomalous complexes (RVACs) (Hartnett et al., 1996). Based on their clinical observation of 143 AMD eyes with intraretinal neovascularization (IRN), Yannuzzi et al. (2001) named this disease as RAP to suggest an intraretinal origin and proposed a three-stage model of progression including IRN (stage 1), subretinal neovascularization (SRN; stage 2), and CNV (stage 3). Gass et al. (2003) proposed a different explanation and suggested the term occult chorioretinal anastomosis (OCRA) to emphasize the choroidal origin of the intraretinal complex. Lacking a definitive sequential histopathologic evidence of its intraretinal vs. choroidal origin, in 2008, type 3 neovascularization was proposed for this entity (Freund et al., 2008) to emphasize the intraretinal location of the vascular complex and distinguish this type from type 1 and type 2 CNV previously described (Gass, 1997) rather than the clinically debated origins (Yannuzzi et al., 2008). MNV3 is the consensus term for this disease entity now (Spaide et al., 2020).

### Epidemiology of Type 3 Macular Neovascularization

Type 3 macular neovascularization accounts for 15–20% of nAMD patients in white populations (Yannuzzi et al., 2008) and 4.5–11.1% among Asians (Song et al., 2009). When dye-based angiography and optical coherence tomography (OCT) were combined to identify lesion composition, MNV3 was found to be the presenting lesion type in 34.2% of eyes with newly diagnosed nAMD (Jung et al., 2014; Li et al., 2018). MNV3 usually occurs in individuals older than 75 years and is popular in women; the male-to-female ratio is about 1:2 (Marticorena et al., 2011; Tsai et al., 2017). There is a tendency toward bilateral involvement. It was found that 40% of the patients with unilateral MNV3 developed an MNV3 lesion in the fellow eye by 1 year, 56% by 2 years, and 100% by 3 years (Gross et al., 2005). The risk of fellow-eye involvement in MNV3 patients is significantly higher than that in typical nAMD patients (Yannuzzi et al., 2001).

### Clinical Features of Type 3 Macular Neovascularization

The main clinical signs of MNV3 include superficial intraretinal hemorrhages and edema, hard exudates, pigment epithelial detachment (PED), and reticular pseudodrusen (RPD) (Maruko et al., 2007; Berenberg et al., 2012; Ueda-Arakawa et al., 2013; Kim et al., 2014, 2015; Ravera et al., 2016; Tsai et al., 2017). The combination of intraretinal hemorrhages, hard exudates, and PED is strongly associated with the presence of a connection between the retinal vasculature and the neovascular complex (Donati et al., 2006). The presence of small retinal hemorrhages, macular soft drusen, and RPD is highly predictive of MNV3, and especially intraretinal hemorrhages are a distinguishing feature from typical CNV (Yannuzzi et al., 2001; Kim et al., 2014; Tsai et al., 2017). RPE atrophy, GA, and focal hyperpigmentation are common features in the fellow eyes of MNV3 (Martins et al., 2018). RPD and a large area of soft drusen are risk factors for bilateral MNV3 (Miki Sawa et al., 2014; Marques et al., 2015; Chang et al., 2016).

### Classification and Multimodal Imaging of Type 3 Macular Neovascularization

The three-stage classification proposed by Yannuzzi et al. (2001) is the most commonly used classification in clinical studies of MNV3, mainly based on clinical findings, fluorescein angiographic (FA), and indocyanine green angiographic (ICGA) findings (Yannuzzi et al., 2001; Tsai et al., 2017). FA revealed a feeding retinal arteriole dipping toward the RPE, forming “an angiomatous lesion” in the subretinal space. The FA features include intraretinal and subretinal leakage with indistinct margins or a vascularized PED, which simulates an occult (type 1) CNV pattern. With ICGA, the MNV3 is seen as a focal area of intense hyper-fluorescence corresponding to the neovascularization (“hot spot”). There is a late extension of the leakage within the retina from the IRN. Recently, multimodal imaging is also being widely applied in the diagnosis and classification of MNV3, including spectral domain optical coherence tomography (SD-OCT) and OCT angiography (OCT-A) (Ravera et al., 2016; Su et al., 2016).

Optical coherence tomography indicates that MNV3 is a focal hyperreflective lesion in the neurosensory retina with surrounding serous fluid (Brancato et al., 2002; Tsai et al., 2017). On high-resolution SD-OCT, the precursor lesion of MNV3 is punctate hyperreflective foci (HRF) in the outer retina (Nagiel et al., 2015). HRF represents two cell types, RPE cells that migrated into the outer retina and lipid-filled cells (Nagiel et al., 2015; Querques et al., 2015; Li et al., 2018). The IRN corresponded to a hyperreflective mass from the outer plexiform layer to the deeper layers and generally originated outside the fovea avascular zone (Matsumoto et al., 2010). The hyperreflective lesion often developed into sub-RPE within underlying drusen or drusenoid PED (Querques et al., 2013). These findings confirm the intraretinal localization of the MNV3, which is always associated with impressive exudative phenomena such as fluid, RPE elevation, and PED. Disruption of the external limiting membrane (ELM) and intraretinal edema internal to the PED are also common in MNV3 (Tsai et al., 2017). OCT-based classification suggested that the origin of MNV3 is from the deep retinal vascular plexus, followed by a disruption of outer retinal layers and penetration through the RPE (Su et al., 2016).

Spectral domain optical coherence tomography also revealed that the subfoveal choroid of MNV3 lesion is significantly thinner than that of age-matched control eyes (Yamazaki et al., 2014). Subfoveal choroidal thickness is considered a predictor of visual outcome and treatment response after anti-vascular endothelial growth factor (VEGF) treatment for typical exudative AMD. A thick choroid was correlated with a better treatment response (Kang et al., 2014). Anti-VEGF therapy on MNV3 can reduce the choroidal thickness significantly for a short time, and a thick choroid has been associated with a higher rate of recurrence of MNV3 (Kim et al., 2016). Thus, OCT is particularly suitable in planning the treatment of MNV3 and monitoring the disease, especially in the context of anti-VEGF therapy (Politoa et al., 2006; Fleckenstein et al., 2021).

Optical coherence tomography angiography is a non-invasive tool and provides independent analysis of blood flow based on motion contrast in the various retinal and choroidal layers (Fingler et al., 2008; Spaide et al., 2015). High-resolution volumetric blood flow information can be obtained to generate angiographic images in a matter of seconds, but no information on vascular wall integrity can be obtained; thus, OCT-A allows a detailed characterization and detection of MNV3, as the vessel structure is not obscured by dye leakage or dye staining of drusen (Perrott-Reynolds et al., 2019). OCT-A illustrates MNV3 lesions as distinct high-flow, tuft-like capillary networks (Borrelli et al., 2018). In the early stage of MNV3, there are frequently small claw-like lesions, which represent the sub-RPE neovascular tissues, connecting to high-flow, tuft-like lesions (Miere et al., 2015). In some cases, a “feeding” vessel can be observed in the neovascular complexes that communicated with inner retinal circulation (Kuehlewein et al., 2015).

Hyperreflective foci on structural SD-OCT represents a precursor lesion of MNV3 (Su et al., 2016). The relationship between HRF on SD-OCT and flow on OCT-A had also been studied. It was demonstrated that HRF on structural OCT corresponds to a small tuft of vessels on OCT-A but only after the development of intraretinal edema, a sign of active MNV3 (Kuehlewein et al., 2015; Tan et al., 2017). However, for nascent MNV3 lesions, detectable flow on OCT-A corresponded to intraretinal HRF on SD-OCT, although no signs of active MNV3 (i.e., intraretinal fluid or serous PED) were noted (Sacconi et al., 2018). Surprisingly, a recent observation suggested that intraretinal edema is not a sign of active MNV3. In that study, the fellow eyes of MNV3 patients had been monitored by structural SD-OCT and OCT-A. It was found that macular edema could occur before neovascularization, and in eyes with MNV3, there was widespread edema with a greater area than that of neovascularization. The intraretinal edema before the formation of MNV3 lesion may be related to VEGF-mediated retinal vessel leakage (Spaide, 2019). Thus, the relationship between flow on OCT-A, HRF, and intraretinal fluid needs further investigation.

### Histopathological Studies of Type 3 Macular Neovascularization

Surgically excised neovascular membranes from MNV3 eyes (a total of 15 specimens) were histopathologically analyzed in two reports (Lafaut et al., 2000; Shimada et al., 2006). It was found that neovascularization was growing out of the neuroretina into the subretinal space (Lafaut et al., 2000). The neovascular masses expressed VEGF and included macrophages and RPE cells. VEGF was also expressed in retinal vessels above the RPE and fibroblasts below the RPE. Hypoxia-inducible factors (HIF-1a and HIF-2a) were expressed in vascular endothelial cells and macrophages (Shimada et al., 2006). None of the cases of stage II lesions showed vascular connections to the choroid, which only was observed in stage III lesions (Shimada et al., 2006). These findings confirm that the initial lesion of MNV3 is IRN, which advances into the sub-RPE space and forms retinochoroidal anastomoses (RCAs).

Postmortem histopathological study of both eyeballs with MNV3 from an 87-year-old woman showed intraretinal vascular complexes in the outer retina and adjacent to the inner portion of the Bruch membrane. The complex had a circumscribed mass of endothelial cells and was surrounded by an eosinophilic matrix. The RPE cells enveloped the lesion. Cells of the angiomatous lesion expressed VEGF, although not as strong as the adjacent neurosensory retina. The RPE was also strongly positive for VEGF, but the choroid exhibited little VEGF expression (Monson et al., 2008; Klein and Wilson, 2011). Similar retinal glomerular angiomatous lesions with encapsulation are also reported in two MNV3 eyes with hematoxylin and eosin (H&E) staining (presented in Figure 1-supplement 4 of Luo et al., 2013). In one eye with MNV3, the glomerular IRN lesion had decreased expression of soluble vascular endothelial growth factor receptor 1 (sVEGFR1) (Luo et al., 2013). Interestingly, the above described retinal glomerular angiomatous lesion with encapsulation in four MNV3 eyes is similar to the concentric layers of proliferating endothelial cells induced by intravitreal injections of VEGF in the eyes of adult primates (Tolentino et al., 1996). It is also similar to the pathological structures of von Hippel–Lindau (VHL) gene mutation-related retinal capillary hemangioblastoma (RCH), which mainly have proliferating-endothelial cells and VHL-deficient foamy (lipid-filled) stromal cells (Park and Chan, 2012).

Postmortem histopathological features of an eye with MNV3 from a 93-year-old man treated with serial ranibizumab injections revealed a vascular complex located from the inner plexiform layer to the inner portion of the Bruch membrane. There were only sparse cells present within the structure of the complex. The RPE monolayer underlying the lesion was disrupted; however, the inner portion of the Bruch membrane was intact. The histopathologic findings corresponded to an area of hyperreflectivity on SD-OCT imaging, confirming the reported tomographic appearance of MNV3 lesions (Skalet et al., 2017).

Serial anti-VEGF treatment likely causes the decreased cellularity in the MNV3 lesion. Postmortem histopathological study of an eye with MNV3 from an 86-year-old woman treated with six intravitreal injections of bevacizumab revealed similar results (Li et al., 2018). High-resolution epoxy resin histologic results indicated that the retinal vascular complex was implanted into the sub-RPE basal laminar deposit. No connection between the choriocapillaris and the sub-RPE space was observed. Both RPE-derived and lipid-filled cells were correlated with clinical intraretinal hyperreflective foci. The sub-RPE space contained macrophages, lymphocytes, Müller cell processes, and subducted RPE cells (Li et al., 2018). The lipid-filled cells are similar to foamy cells found in RCH, which may indicate deregulated lipid metabolism and the VHL–HIF hypoxia pathway (Park and Chan, 2012; Wei et al., 2019).

These pathological studies are consistent with the multimodal imaging findings (Borrelli et al., 2018) and reinforce the proposed retinal (but not choroidal) origin of MNV3 and suggest the involvement of retinal hypoxia, inflammation, and multiple cell types (such as photoreceptors, Müller cells, RPE cells, macrophages, microglial cells, lipid-filled cells, and fibroblasts) in the development and progression of MNV3. The expression of VEGF/HIFs in the MNV3 lesion and/or surrounding cells (Shimada et al., 2006), decreased expression of sVEGFR1 in the MNV3 lesion (Luo et al., 2013), the structure of glomerular IRN (Monson et al., 2008; Klein and Wilson, 2011; Luo et al., 2013), and the existence of intraretinal lipid-filled cells beside IRN (Li et al., 2018) support the idea that the VHL–HIF–VEGF hypoxia pathway mediates the pathogenesis of MNV3.

### Anti-vascular Endothelial Growth Factor and Anti-inflammation Therapy of Type 3 Macular Neovascularization

Before the advent of intravitreal anti-VEGF therapy, treatment outcomes were generally poor. Most MNV3 patients declined to 20/200 or worse visual acuity because of subretinal fibrosis or disciform scar formation (Bottoni et al., 2005). Thus, MNV3 was considered a particularly treatment-resistant form of AMD (Scott and Bressler, 2009), which may not be true now.

Intravitreal injection of triamcinolone acetonide (IVTA) in combination with photodynamic therapy (PDT) or focal laser photocoagulation is an option for treating retinal neovascularization before the advent of anti-VEGF therapy. Triamcinolone can suppress the inflammation cascade and reduce intraretinal vascular leakage. PDT/IVTA treatment had been used in many clinical studies treating MNV3, especially for stages II and III MNV3 (Gupta et al., 2010). The results were generally promising, as many eyes had stable or improved visual acuity or had complete resolution of vessel leakage (Freund et al., 2006; Montero et al., 2009). A study with a 3-year follow-up compared the therapeutic effects between anti-VEGF alone, PDT with anti-VEGF, and PDT with IVTA treatment of MNV3. Vision improved only in the group treated with PDT/IVTA, although the group treated with PDT/anti-VEGF had the best anatomical outcome. However, more patients who received IVTA developed GA and underwent cataract surgery (Rouvas et al., 2012).

Anti-VEGF therapy has achieved favorable outcomes in MNV3, especially if treatment was initiated during the early stage of the disease (stages I and II). Early diagnosis of MNV3 is possible using multimodal imaging such as OCT/OCT-A now. For instance, 1 year after anti-VEGF treatment, the communication between deep retinal capillaries and the RPE/sub-RPE space on OCT-A disappeared, while the neovascular tuft persisted at the level of the deep retinal capillaries (Miere et al., 2017; Sacconi et al., 2021). Anti-VEGF treatment induced about 70% complete occlusion of the lesion at 2–3 years, and stable or improved visual acuity was obtained in 95% and 100% of eyes at 2 years and 3 years, respectively (Gharbiya et al., 2014).

However, anti-VEGF therapy can induce the development of GA and RPE rip. The long-term visual outcome of stage III lesions is still poor (Daniel et al., 2016), and recurrences are common after the cessation of treatment (Baek et al., 2016). In stage III, the lesion showed persistent leakage after treatment, and the rate of lesion occlusion is low (Malamos et al., 2018). Patients with MNV3 who had no lesion on an OCT-A scan after anti-VEGF treatment showed a lower recurrence rate and maintained visual acuity with fewer injections than those with persistent high-flow lesions on an OCT-A scan (Han et al., 2020). The incidence rate of massive subretinal hemorrhage increased steadily with time for stage III MNV3 (Lee et al., 2017; Han et al., 2020). The early sign of recurrence is detectable flow deepening from the deep retinal capillaries to the RPE/sub-RPE space using OCT-A (Sacconi et al., 2021).

## Animal Models of Type 3 Macular Neovascularization

While epidemiological, pathological, multimodal imaging and therapeutic studies in humans have provided important clues toward the potential causes of MNV3, elucidation of its underlying mechanisms has only been possible using experimental animal models. Based on the pathological studies, retinal hypoxia and inflammation are obviously involved in the development of MNV3 through multiple cell types. Currently, there are seven major types of mouse model commonly used in the field, including three models directly related to the hypoxia pathway (retina-specific *VhlKO* mice, *Vegf* overexpression, and *sVEGFR1* knockout in mouse photoreceptors), two models related to abnormal lipid metabolism (*Vldlr^–/–^* mice and *Cyp27a1^–/–^* mice), one model related to cell migration pathway (endothelial cell-specific *Srf* knockout mice), and RNV3 (JR5558) mice that have mutations in genes regulating retinal polarity and inflammation (Table 1).

**TABLE 1:** Animal models of MNV3.

	Mouse models	Genes	Target cells	Vascular origin	IRN	SRN	RCA	Photoreceptor degeneration	References
1	*alpha-Cre,VhlKO*	Vhl	Retinal progenitors	Retina	a few	NO	NO	Yes	Kurihara et al., 2010; Lange et al., 2011a
2	*Chx10-Cre,VhlKO*	Vhl	Retinal progenitors	Retina	a few	NO	NO	Yes	Wert et al., 2016
3	*alpha-Cre,Rb/p107/VhlTKO*	Rb/p107/Vhl	Retinal progenitors	Retina	Yes	Yes	Yes	Yes	Wei et al., 2019
4	*Opsin-Cre, VhlKO*	Vhl	Rods	Retina	NO	NO	NO	Yes	Lange et al., 2011b
5	*Pde6g-CreERT2, VhlKO*	Vhl	Rods	Retina	Yes	NO	NO	Yes	Zhang et al., 2019
6	*BP-Cre, VhlKO*	Vhl	Cones	Retina	Yes	NO	NO	Yes	Barben et al., 2018b
7	*Trp1-Cre, VhlKO*	Vhl	RPE cells	Retina	Yes	Yes	Yes	Yes	Lange et al., 2012
8	*rho/VEGF*	VEGF	Rods	Retina	Yes	Yes	NO	Yes	Tobe et al., 1998; Ohno-Matsui et al., 2002
9	*iCre-75, Vegfr1KO*	VEGFR1	Rods	Retina	Yes	Yes	NO	Yes	Luo et al., 2013
10	*Vldlr^–/–^*	Vldlr	all Cells	Retina	Yes	Yes	Yes	Yes	Heckenlively et al., 2003
11	*Cyp27a1^–/–^*	Cyp27a1	all Cells	Retina/Choroid	Yes	Yes	Yes	NO	Omarova et al., 2012
12	*Srf^–/–^*	Srf	Adult endothelial cells	Retina	Yes	Yes	NO	Yes	Weinl et al., 2013
13	*RNV3 (JR5558)*	Crb1/Jak3	all Cells	Retina	Yes	Yes	NO	Yes	Hasegawa et al., 2014; Nagai et al., 2014; Chang et al., 2018

### Retina-Specific *Vhl* Knockout Mice With or Without *Rb* Gene Knockout

As discussed above, the expression of VEGF/HIFs in MNV3 lesions and surrounding cells (Shimada et al., 2006) and the decreased expression of sVEGFR1 in MNV3 lesions (Luo et al., 2013) support the idea that the VHL–HIF–VEGF hypoxia pathway mediates the pathogenesis of MNV3. HIF is the master regulator of the response to hypoxia because HIF-α induces the transcription of genes involved in hypoxia adaptation, including erythropoietin (EPO) and VEGF (Schofield and Ratcliffe, 2004; Semenza, 2004). VHL tumor suppressor protein is a component of the E3 ubiquitin ligase complex that targets HIF-α for proteasomal degradation. Human VHL gene mutations cause VHL disease, a multisystem tumor syndrome associated with many tumors, such as brain hemangioblastoma and clear cell renal cell carcinomas (CCRCCs) (Lonser et al., 2003). RCH is the hallmark lesion of ocular VHL disease (Park and Chan, 2012). Histologically, RCH mainly consists of vascular endothelial cells and vacuolated “foamy” lipid-filled stromal cells likely due to VHL deficiency-induced abnormal lipid metabolism, also seen in the CCRCC and liver hemangioblastoma (Haase et al., 2001). The foamy stromal cells are considered the actual tumor cells in the hemangioblastoma (Park and Chan, 2012). These histological features of RCH are similar to the pathological finding of retinal specimens from MNV3 patients, including glomerular IRN (Monson et al., 2008; Klein and Wilson, 2011; Luo et al., 2013) and intraretinal lipid-filled cells beside IRN (Li et al., 2018).

Several retina-specific *Vhl* knockout mice have been established using different Cre mouse lines and *Vhl* floxed mice. These studies shed great light on the action of the hypoxia pathway in the retina and elucidate many aspects of the pathogenesis of MNV3. Three studies using *Pax6 α-Cre* or *Chx10-Cre* to knockout (KO) *Vhl* in retinal progenitors found that *VhlKO* delays the regression of hyaloid vessels and the development of retinal vessels (Kurihara et al., 2010; Lange et al., 2011a) or prompt retinal capillary dropout (Wert et al., 2016). Retinal vessel density was notably reduced in the Cre-expression area, while a few vessels were penetrating the outer nuclear layer (ONL) (Kurihara et al., 2010; Lange et al., 2011a). Both *Pax6 α-Cre* and *Chx10-Cre*-mediated *VhlKO* retinas have upregulated Hif expression, severe retinal degeneration, and reduced electroretinogram (ERG) response. While these phenotypes resemble the early stage of human MNV3, paradoxically, high Hif does not induce extensive angiogenesis as expected but instead inhibits retinal angiogenesis in the murine *Vhl* null retina. Deleting *Vhl* in all retinal cells and vascular endothelial cells using *UBC-Cre^*ER*^* also suppresses retinal angiogenesis (Arreola et al., 2018). The mechanism is unknown, but we figured this out (Wei et al., 2019).

We showed that retinoblastoma tumor suppressor (Rb) constrains the expression of some Hif target genes in the *Vhl^–/–^* retina. Deleting *Rb* induced extensive retinal neovascularization and autophagic ablation of photoreceptors in the *Vhl^–/–^* retina. Unexpectedly, triple knockout (*Rb/p107/Vhl*TKO) mice do not develop retinoblastoma as expected (Chen et al., 2004) but generate subretinal vascular growths resembling MNV3 and RCH (Wei et al., 2019). The subretinal MNV3/RCH-like lesions broke the RPE and Bruch membrane to form RCA. Most stromal cells in the MNV3/RCH-like lesions were Sox9^+^, suggesting a Müller glia origin, and expressed Gal3, a marker of human brain hemangioblastoma (Al-Salam et al., 2013). RNA sequencing revealed that the complement pathway, extracellular matrix pathway, and phosphoinositide 3-kinase (PI3K) pathway are activated (Wei et al., 2019). This model has important implications in understanding the pathogenesis of MNV3, as this model mimics some important environmental risk factors such as smoking, which can inactivate the *Rb* gene (Dasgupta et al., 2006), and retinal hypoxia, which can inactivate the *Vhl* gene (Lonser et al., 2003).

*Ptf1a-Cre*-mediated *VhlKO* in amacrine and horizontal cells induced overdevelopment of deep and intermediate vascular plexuses without neovascularization (Usui et al., 2015). Two studies knocked out the *Vhl* gene in rod cells using *opsin-Cre* (Lange et al., 2011b) or *Pde6g-CreERT2* mice (Zhang et al., 2019). *Opsin-Cre-*induced photoreceptor hypoxia had no noticeable retinal vascular changes but had retinal degeneration at a late stage (Lange et al., 2011b; Barben et al., 2018a). Tamoxifen-induced *CreERT2*-mediated photoreceptor hypoxia can cause IRN/SRN, resembling the early stage of human MNV3 (Zhang et al., 2019). Knocking out *Vhl* in cone cells using *cone-opsin Cre (BP-Cre)* and *R91W;Nrl^–/–^* mice demonstrated that cone hypoxia could cause IRN, resembling early MNV3 (Barben et al., 2018b). All major phenotypes of *VhlKO* in retinal progenitors, amacrine and horizontal cells, rod, and cones can be rescued by *Hif1a* knockout, but not *Hif2a* knockout (Kurihara et al., 2010; Usui et al., 2015; Barben et al., 2018a; Barben et al., 2018b). Knocking out *Vhl* in RPE cells using *Trp1-Cre* induced IRN, SRN, and RCA, which cannot be rescued by *Hif1a* knockout, indicating these MNV3-like lesions are mediated by *Hif2a* (Lange et al., 2012).

These *VhlKO* models confirm the importance of retinal hypoxia, especially hypoxia of outer retinal cells, including photoreceptors and RPE cells, in the pathogenesis of MNV3. The Rb–E2F pathway can modulate the hypoxia response by regulating the expression of some Hif target genes. Only the *Rb/p107/Vhl TKO* model and RPE-specific *VhlKO* model can induce stage III MNV3.

### *Rho/VEGF* and *IRBP/VEGF* Mice

Transgenic mice with bovine rhodopsin promoter-driven human VEGF165 expression in the photoreceptors (*rho/VEGF* mice) show focal areas of IRN and SRN originating from the deep capillary bed of the retina and vascular leakage (Okamoto et al., 1997). The *VEGF* transgene mRNA begins to express at postnatal day 6 (P6) and reaches a constant level between P14 and P21, but VEGF protein is only detected in some photoreceptors in focal areas. IRN lesions form at P14, and abnormal vessels reach the subretinal space by P18. In adult *rho/VEGF* retinas, blood vessels extend from the inner nuclear layer toward RPE to form an extensive plexus in the subretinal space. The SRN lesions are progressively engulfed by the RPE (Tobe et al., 1998).

Another doxycycline-inducible version of this transgenic mouse was also generated, including *rtTA-rho/TRE-VEGF* and *rtTA-IRBP/TRE-VEGF* mice (Ohno-Matsui et al., 2002). At 3 weeks–6 months of age, mice were treated with doxycycline in their drinking water or by daily subcutaneous injection. Doxycycline treatment induced IRN/SRN and tractional retinal detachment. The incidence of these phenotypes and their severity depended on doxycycline dosage (Ohno-Matsui et al., 2002).

These models, which present with features that mimic MNV3, demonstrate that overexpression of VEGF in photoreceptors is enough to induce IRN/SRN rather than CNV as RPE and Bruch membrane are intact. These models are similar to the rod/cone-specific *VhlKO* models (Barben et al., 2018b; Zhang et al., 2019). They are useful tools for investigating VEGF-induced early changes in the retina and are valuable models to test the effects of some angiogenesis inhibitors on retinal neovascularization (Liu et al., 2017).

### Photoreceptor-Specific *VEGFR1* Knockout Mice

Retinal neurons can limit angiogenesis by titrating VEGF for vascular endothelial cells by expressing VEGFR1 and VEGFR2 (Luo et al., 2013; Okabe et al., 2014). Reducing the expression of these receptors in retinal neurons may increase the available VEGF molecules for retinal vascular endothelial cells, thus inducing retinal neovascularization.

VEGFR1 (also called FLT-1) is widely expressed in many types of cells, including endothelial cells and neurons, and has diverse biological functions in different cell types. VEGFR1 is alternatively spliced to yield both soluble (sVEGFR1) and membrane-tethered (mVEGFR1) isoforms. Both isoforms bind VEGF-A with 10-fold higher affinity than VEGFR2, but mVEGFR1 has weak kinase activity in endothelial cells (Boucher et al., 2017). VEGFR1 acts as a decoy receptor by binding VEGFA and reducing VEGFR2 signaling (Gille et al., 2000); sVEGFR1 or sFLT-1 also saturates VEGFA and reduces VEGFA binding to VEGFR2 (Kendall and Thomas, 1993). VEGFR1 is therefore considered to be a negative regulator of angiogenesis due to its negative regulation of VEGFR2 signaling. *VEGFR1^–/–^* mice die at the embryonic stage, associated with aberrant endothelial cell proliferation and formation of disorganized vessels with partially obstructed lumens (Simons et al., 2016). Endogenous sVEGFR1 is both necessary and enough for corneal avascularity (Ambati et al., 2006). Retinal photoreceptors can express sVEGFR1 (Luo et al., 2013); thus, knocking out *sVEGFR1* in rods may free VEGF to promote angiogenesis. The *iCre-75* mouse is a rod-specific Cre transgenic mouse line, in which 4-kb mouse rhodopsin gene promoter drives the expression of Cre recombinase in rods (Li et al., 2005). Indeed, *Vegfr1 floxed/iCre-75* mice develop MNV3-like lesions at 1–3 months of age, with a penetrance of about 50–60% (Luo et al., 2013).

Vegfr2 is more abundantly expressed in retinal neurons than in retinal endothelial cells during the first week after birth but gradually decreases as development continues; at P13, only some Müller cells still express Vegfr2 (Okabe et al., 2014). Vegfr2 on retinal neurons can bind and engulf VEGF proteins around neurons. In neuron-specific deletions of *Vegfr2 (Pax6 α-Cre, Vegfr2 floxed)*, free VEGF proteins are markedly increased due to neurons insufficiently engulfing VEGF, which result in misdirected vertical angiogenic growth toward neurons and dense intraretinal vascular plexus. However, this mutant mouse has no MNV3 lesion (Domigan and Iruela-Arispe, 2014; Okabe et al., 2014). A major difference between the *sVEGFR1* deletion model (Luo et al., 2013) and the *Vegfr2* deletion model (Okabe et al., 2014) is the Cre mouse line, iCre-75 only expresses in rods, but Pax6 α-Cre can express in all retinal cells (Marquardt et al., 2001). These two *VEGFR* deletion models suggest free VEGF proteins around rods, but not all retinal neurons are critical for MNV3 lesion formation. It would be interesting to see if rod-specific deletion of *Vegfr2* can cause MNV3-like lesions.

### Very Low-Density Lipoprotein Receptor Null (*Vldlr^–/–^*) Mice

The very low-density lipoprotein receptor (*Vldlr*) gene is one of the functional candidate genes for a significant association with AMD in humans (Haines et al., 2006). Vldlr is an 86-kDa transmembrane protein that belongs to the low-density lipoprotein receptor (Ldlr) family. Vldlr is highly expressed in photoreceptors and can be detected in RPE cells and retinal vascular endothelial cells (Hu et al., 2008; Dorrell et al., 2009; Joyal et al., 2016).

Very low-density lipoprotein receptor null mice (B6;129S7-Vldlr^*tm1Her*^/J, Jax Stock No.: 002529) have been shown to develop retinal neovascularization starting at ∼2 weeks of age with 100% penetrance but do not develop dyslipidemia. Normal and heterozygous mice do not have this phenotype (Frykman et al., 1995; Heckenlively et al., 2003; Hu et al., 2008). The progression of retinal lesions resembles human MNV3, including the three stages of IRN, SRN, and CNV or RCA. As early as P12, neovascular vessels from the outer plexiform layer grow into the avascular zone of the outer nuclear layer. From P15–P18, the new vessels gradually extend into the subretinal space. Focal RPE detachment was present in the early stage of SRN. RPE activation and subretinal fibrosis at lesion sites are in the late stage of SRN (Hu et al., 2008). Subretinal hemorrhages are evident by age ∼2 months. However, breaks in the Bruch membrane and RCA are not observed in mice less than 6 months of age, confirming the intraretinal origin of subretinal NV. RCA, secondary photoreceptor degeneration, and regression of the retinal neovascularization can be observed by age ∼8 months (Heckenlively et al., 2003; Li et al., 2007; Hu et al., 2008; Dorrell et al., 2009; Chen et al., 2020).

Fluorescein leakages are first noticeable at 3 weeks of age, and the number of leakage spots continues to increase with age and reaches a maximum level by 6 weeks. Leakage spots begin to reduce significantly after ∼8 months of age and are hardly noticeable at 12 months because of subretinal fibrosis or scarring (Heckenlively et al., 2003; Li et al., 2007; Hu et al., 2008). Although the numbers of subretinal sprouts are reduced in older mice, most intraretinal and subretinal vessels persist throughout the life of the *Vldlr^–/–^* mice (Dorrell et al., 2009). The morphology of the neovascular growths transits from initial vascular buds to balloon-shaped bulbs and then to mature angiomatous-like vascular tangles, reminiscent of the glomerular IRN found in postmortem human MNV3 lesions (Hu et al., 2008; Monson et al., 2008; Klein and Wilson, 2011; Luo et al., 2013).

Very low-density lipoprotein receptor null*^–^* vascular lesions are associated with Müller glia activation at late stages, as demonstrated by glial fibrillary acidic protein (GFAP) expression, retinal rosette formation, and altered growth factor expression. VEGF and basic fibroblast growth factor (bFGF) expression increases in the photoreceptor layer around the IRN lesion from ∼4 weeks of age (Li et al., 2007). *Vldlr^–/–^* mice also develop chronic inflammation in the retina and RPE (Li et al., 2007; Chen et al., 2009; Dorrell et al., 2009). The increased VEGF levels observed in *Vldlr^–/–^* mice are initially suggested to be linked to the negative regulatory role of Vldlr on Wnt signaling, which targets VEGF (Chen et al., 2007). However, since Vldlr has important functions in cholesterol homeostasis, lipid metabolism, and transport (Tiebel et al., 1999), it is demonstrated that *AAV2-GRK1-shVldlr-*mediated *Vldlr* gene knockdown in photoreceptors alone can develop MNV3-like lesions, suggesting that photoreceptors can not only get energy from glucose but also use fatty acid (FA) β1 oxidation (Joyal et al., 2016). It is demonstrated that impaired fatty acid uptake in *Vldlr^–/–^* mice results in a reduction of α-ketoglutarate, which decreases prolyl hydroxylase dehydrogenase activity and promotes the stabilization of Hif-1a. Subsequently, VEGF expressions increase in photoreceptors (Joyal et al., 2016). Thus, dysregulated energy metabolism might drive pathological neovascularization by stabilizing Hif1a and overexpression of VEGFA in *Vldlr^–/–^* photoreceptors.

Very low-density lipoprotein receptor is also the receptor of reelin and is essential for reelin signaling in neuronal migration and synaptic plasticity (Lane-Donovan and Herz, 2017). In *Vldlr^–/–^* mice, mistargeted neurites of horizontal cells act as a scaffold for growing neovascularization (Johnson et al., 2015). VEGFA produced by RPE cells in *Vldlr^–/–^* mice can induce subretinal infiltration of microglia/macrophages, which can migrate into the ONL and promote the formation of subretinal NV (Usui-Ouchi et al., 2020). Thus, overexpression of VEGFA in photoreceptors and RPE cells, mistargeted neurites of horizontal cells, and activated microglial cells are the three major mechanisms of retinal neovascularization in *Vldlr^–/–^* mice, which represent the function of Vldlr in lipid metabolism and reelin signaling, respectively.

The unique pathological features in the *Vldlr^–/–^* mouse model enable its wide use to investigate the potential mechanisms and treatments of retinal neovascularization, including antioxidants, neurotrophic factors, resveratrol, anti-inflammation, cellular bioenergetics, and transcriptional control (Dorrell et al., 2009; Joyal et al., 2016; Liu et al., 2017). For instance, the role of some pro-inflammatory factors involved in the development of MNV3 was investigated in the *Vldlr^–/–^* mouse. The expression of intercellular adhesion molecule (ICAM)-1, interleukin (IL)-18, tumor necrosis factor (TNF)-α, endothelial nitric oxide synthase (eNOS), and cyclooxygenase (COX)2 significantly increased in *Vldlr*^–/–^ mouse retina and RPE (Li et al., 2007; Chen et al., 2009; Dorrell et al., 2009). Interestingly, the expression level of the suppressor of cytokine signaling 3 (SOCS3) decreased significantly in *Vldlr^–/–^* retinas, and daily SR1001 treatment increased the retinal Socs3 expression and significantly inhibited the MNV3 phenotypes (Sun et al., 2015). Furthermore, the expression of the master inflammatory regulator c-Fos increased in *Vldlr^–/–^* photoreceptors, which induced IL-6 and TNF expression, activated signal transducer and activator of transcription (STAT)3 and increased TNF-α-induced protein 3 (Tnfaip3) expression. Suppressing c-Fos reduced the pathological neovascularization and rescued visual function in *Vldlr^–/–^* mice (Sun et al., 2017; Wang et al., 2020).

### *Cyp27a1^–/–^* Mice

Different cytochrome P450 (Cyp) enzymes play essential roles in maintaining cholesterol homeostasis, including cholesterol biosynthesis and elimination (Pikuleva, 2006). Cyp27a1 is a ubiquitously expressed mitochondrial sterol 27-hydroxylase that catalyzes the hydroxylation of cholesterol and other sterols at position C27 (Pikuleva, 2006). It is widely expressed in the retina and is the principal cholesterol hydroxylase in the retina where it accounts for the majority of enzymatic cholesterol elimination (Mast et al., 2011; Pikuleva and Curcio, 2014).

In humans, Cyp27a1 deficiency leads to cerebrotendinous xanthomatosis (CTX). CTX is a multi-symptom lipid storage disease whose typical clinical features include neurological dysfunction and tendon xanthomas (Björkhem and Hansson, 2010). CTX patients have some ocular abnormalities, such as juvenile bilateral cataracts, cholesterol-like crystals in the vitreous, premature retinal senescence with drusen and retinal vessel sclerosis, and cholesterol-like deposits along the vascular arcades (Cruysberg et al., 1995). Changes in *Cyp27a1^–/–^* mice, however, are less pronounced than those in humans. They lack classic symptoms of CTX, such as cataracts, brain and tendon xanthomas, and atherosclerosis. This may reflect distinct manifestations of sterol 27-hydroxylase deficiency in *Cyp27a1^–/–^* mice and CTX patients (Honda et al., 2001). Nevertheless, *Cyp27a1^–/–^* mice are associated with MNV3-like lesions, including RCA, and vascular leakage and focal depositions containing cholesterol along the Bruch membrane (Omarova et al., 2012).

Spectral domain optical coherence tomography examination reveals 3–10 small hyperreflective spots in the inferior retina of 6-week-old mutant mice. Most spots do not change with age, although some grow in diameters. Simultaneous disturbances in the outer plexiform layer (OPL) and RPE occur in all studied young *Cyp27a1^–/–^* mice. At 11 months old, large hyperreflective spots can be determined, and blends between the retinal and choroidal circulations can be found by the Doppler flow of SD-OCT. By 12 months, 63% of *Cyp27a1^–/–^* females and 84% of *Cyp27a1^–/–^* males have at least 1–2 large hyperreflective spots in both eyes. Histologically, small OPL hyperreflective spots are abnormally dilated blood vessels at 6 weeks old, and OPL disruption and torsion near the inner nuclear layer (INL)/OPL interface at 8 months old. Large lesions represent the formation of RCA involving blood vessels between the INL and choroid at 11 months old. Activated Müller cells can be observed along with the RCA also in the whole retina. Notably, focal lipid depositions along the Bruch membrane, similar to soft drusen and basal linear deposits in AMD, can be found. Pimonidazole labeling and Hif-1a staining suggest retinal and choroidal hypoxia in the *Cyp27a1^–/–^* mice, while the mechanism is still unknown (Omarova et al., 2012).

Unlike *Vldlr^–/–^* mice in which IRN comes from the retina, the neovascularization in *Cyp27a1^–/–^* mice seems to occur in both the retina and choroid. Unlike *Vldlr^–/–^* mice that have an abnormal ERG indicating secondary photoreceptor degeneration, *Cyp27a1^–/–^* mice have a normal ERG response measured at 2–12 months old as the retinal lesions occupy only a tiny retinal area (Chen et al., 2009; Dorrell et al., 2009; Omarova et al., 2012; Joyal et al., 2016). An important difference between human MNV3 and *Cyp27a1^–/–^* mice is that human MNV3 is more popular in women than men (Marticorena et al., 2011; Tsai et al., 2017), but *Cyp27a1^–/–^* males have a higher frequency of retinal/choroidal pathologies than females, as males had a greater elevation in the rate of cholesterol biosynthesis (2.6-fold in males vs. 1.7-fold in females) (Omarova et al., 2012). As such, *Cyp27a1^–/–^* mice have not yet been widely used in the field of MNV3 study.

### *Srf^–/–^* Mouse

Serum response factor (SRF) is a nuclear transcription factor modulating gene expression in concert with cytoskeletal assembly and disassembly, important for motile cell functions, such as cell migration, guided movement, engulfment, adhesion, and contraction (Olson and Nordheim, 2010). Myocardin-related transcription factors (MRTFs), including MRTF-A and MRTF-B, are key coactivators of SRF that link actin dynamics to SRF-mediated gene transcription (Gau and Roy, 2018). The activity of MTRFs is controlled by cytoskeletal actin dynamics, which is regulated by Rho-GTPase signaling (Posern and Treisman, 2006; Weinl et al., 2013). Numerous studies have demonstrated that loss of function of either MRTF or SRF causes defects in the actin cytoskeleton and migration of a diverse range of mammalian cells in certain physiological contexts, such as development, angiogenesis, hematopoiesis, and immune function (Gau and Roy, 2018).

Myocardin-related transcription factors-Serum response factor signaling is essential for angiogenesis and is regulated by VEGF. Endothelial cell (EC)-specific *SRF* deletion leads to aneurysms and hemorrhages from E11.5 and lethality at E14.5. Mutant embryos present a reduced capillary density and defects in EC migration, with fewer numbers of filopodia in tip cells and ECs showing defects in actin polymerization and intercellular junctions. Knockdown of *SRF* in ECs impaired VEGF-induced *in vitro* angiogenesis (Franco et al., 2008; Weinl et al., 2013). SRF is strongly expressed in a subset of ECs in active vascular sprouting regions, with the highest level observed in endothelial tip cells and stalk cells. SRF selectively controls tip cell invasion and filopodia formation but may not be related to vascular remodeling (Franco et al., 2013). SRF promotes the expression of VEGFR2, β-actin, and VE-cadherin, which is vital for the VEGF–VEGFR2 signaling (Franco et al., 2008). On the other hand, VEGF can promote MRTF nuclear accumulation (Weinl et al., 2013; Hinkel et al., 2014). VEGF also promotes SRF expression and increases SRF binding activity to DNA in ECs (Chai et al., 2004).

Pharmacological inhibition of MRTF–SRF signaling by CCG-1423 impedes endothelial cell migration and angiogenesis (Gau et al., 2017). Deletion of *SRF* in postnatal ECs at P1–P4 induces a significant delay in the development of the superficial vascular plexus and absence of deep vascular plexus, resembling major characteristics of inherited human retinal pathologies such as retinopathy of prematurity (ROP) and familial exudative vitreoretinopathy (FEVR), including Norrie’s disease (Wang et al., 2012; Franco et al., 2013; Weinl et al., 2013).

However, *SRF* deletion in ECs induced in adult mice at 4–6 weeks of age triggers unwarranted IRN lesions, reminiscent of the human MNV3 (Weinl et al., 2013). These new vessels originate from retinal deep capillaries and develop non-uniformly distributed focal lesions. These lesions also have a thin ONL with secondary retinal degeneration, rupture of the RPE, lamination defects, and cell displacement within the INL and ONL. Interestingly, the mRNA level of VEGFA is unchanged in this model (Weinl et al., 2013). Chromatin immunoprecipitation (ChIP) experiments and *in vitro* siRNA knockdown prove that the Thbs1 gene, which encodes the Vldlr ligand thrombospondin 1, is a downstream target of SRF. This may partially explain the resemblance of intraretinal new vessels between adult EC-specific SRF deficiency mice and with the *Vldlr^–/–^* mice (Weinl et al., 2013; Joyal et al., 2016).

This adult mouse model with EC-specific SRF deficiency is unique, as there is no change of VEGF levels; it is also known that SRF promotes the expression of VEGFR2, which is vital for the VEGF–VEGFR2 signaling (Franco et al., 2008). Thus, this mouse model induces retinal neovascularization without upregulation of VEGF and VEGFR2. However, that *SRFKO* only induces IRN in adult but not young mouse matches the fact that AMD occurs in old persons. The molecular mechanism and the involvement of Vldlr ligand thrombospondin-1 in this model need to be further studied.

### *RNV3 (JR5558)* Mice

*RNV3* (B6.Cg-Crb1^*rd8*^ Jak3^*m1J*^/Boc, Jax Stock No.: 005558) mice are also known as retinal vascularization 3 model, *NRV2* mice, and *JR5558* mice, which have mutations in the *Crb1* and *Jak3* genes (Chang et al., 2018). It was identified in a B6;129 mixed background strain with unique eye phenotypes through The Jackson Laboratory Eye Mutant Screening program (Won et al., 2011). This model was previously described by two groups (Hasegawa et al., 2014; Nagai et al., 2014). The first report indicated that the ectopic vascular vessels are choroidal in origin and infiltrated the RPE and intraretinal space (Nagai et al., 2014). The second group reported that the model captures the early stages of MNV3, with intraretinal vessels diving into the subretinal space but not breaching the Bruch membrane, and there was no RCA (Hasegawa et al., 2014). Now it is accepted that the lesions originate from the retina (Chang et al., 2018; Borrelli et al., 2020).

The major clinical manifestations are areas of retinal depigmentation. These depigmentation spots can be observed at P18 without fluorescein leakage. The lesions become well-demarcated and larger at P25, and some of the depigmented areas correspond to areas of fluorescein leakage. The retinal depigmented spots and areas of fluorescein leakage increase in number and size with age, with the maximal number of lesions reaching about 20 per eye at about 1 month. The areas of depigmentation correlate with the development of vascular leakage, indicating a vascular component to this disease phenotype. Around 2 months of age, some individual retinal depigmented spots appear to merge, generating larger areas of depigmentation. As with focal depigmentation, fluorescein leakages gradually decrease after P25 (Hasegawa et al., 2014; Nagai et al., 2014; Chang et al., 2018).

Histological studies indicate that new vessels begin to grow from INL at P15, extend into RPE, and form balloon-like vessel structures from P17 to P25. Photoreceptor cell degeneration can be observed where the ectopic vasculature disrupts the retinal structure. The neovascular lesions are enveloped by the RPE in the photoreceptor cell layer and subretinal space by 3 months of age. By 8 months of age, mutants showed a much-reduced rod and cone ERG response compared to that of C57BL/6J wild-type controls. VEGF levels of the RNV3 retina are significantly higher than those of C57BL/6 control mice, and blockade of VEGF-A with a neutralizing monoclonal antibody can reduce the average number and size of neovascularization lesions (Hasegawa et al., 2014; Nagai et al., 2014; Chang et al., 2018). Thus, RNV3 neovascularization originates from the retinal vascular plexus and grows outward to the subretinal space, forming neovascularization structures at the RPE–Bruch membrane interface and mimicking the early clinical presentation of MNV3 in humans (Hasegawa et al., 2014; Chang et al., 2018). The balloon-like vessel structures of this model are similar to the retinal glomerular angiomatous lesions with encapsulation found in MNV3 patients (Monson et al., 2008; Klein and Wilson, 2011; Luo et al., 2013). Interestingly, the retinal new vessels show fenestrations along their contact with RPE, similar to the choriocapillaris (Hasegawa et al., 2014). RPE cells have the ability to induce fenestrations in endothelial cells of retinal vessels by encapsulating them (Burns and Hartz, 1992). Thus, RPE encapsulation is a possible mechanism, but further investigation is needed.

This mutant mouse model appears to have a recessive inheritance (Nagai et al., 2014; Borrelli et al., 2020). Genetic analysis localized *RNV3* to mouse chromosome 1 in a region containing the *Crb1* gene, *Cfh* (component factor h) gene, and Cfh-related genes 1 through 3 (*Cfhr1–Cfhr3*). High throughput whole-exome sequencing identified a single base deletion in the *Crb1* (crumbs family member 1) gene, which was previously reported to cause retinal degeneration 8 (*rd8*); no coding sequence variation was detected for *Cfh* and *cfhr1–cfhr3* genes (Mehalow et al., 2003; Nagai et al., 2014; Chang et al., 2018). The transcription activator-like effector nuclease (TALEN)-mediated oligonucleotide-directed repair (ODR) of this *Crb1* gene deletion rescued the posterior segment vascularization phenotype, indicating that *Crb1* mutation is necessary for the MNV3-like lesions in this mouse model (Low et al., 2014; Chang et al., 2018). Whole-exome sequencing also identified *Jak3*^*m1J*^ gene mutation; however, this mutation alone cannot cause the MNV3-like lesions. When combined with the disruption in the *Crb1* gene, it can enhance the retinal vascular phenotype (Chang et al., 2018).

Crb1 is a membrane protein that establishes cell polarity and adhesion between cells. Crb1 is expressed in Müller glia cells and contributes to adherens junctions that constitute the external limiting membrane (ELM). The *Crb1*^*rd8*^ mutant mice have retinal ELM fragmentation and outer retinal dysplasia but no new vascular lesions (Mehalow et al., 2003). The phenotypic difference between *Crb1*^*rd8*^ and *RNV3* is most likely due to genetic background differences between the strains, for instance, the *Jak3* mutation in *RNV3* mice. Janus kinase 3 (Jak3) is critical for the normal development and function of the immune system, including B cells, T cells, and natural killer cells, thus affecting inflammation responses (Thomis et al., 1995). So, retinal polarity defects combined with abnormal inflammation response may drive the MNV3-like lesions in this model.

This model is a valuable platform to study the pathogenesis of MNV3, as well as for evaluating potential therapeutics, such as C-C chemokine receptor type 3 antagonists, simultaneous VEGF-A/ANG-2 neutralization, and DICER1 variant (OptiDicer) (Nagai et al., 2015; Foxton et al., 2019; Wright et al., 2020; Joussen et al., 2021). For instance, CCR3 is a receptor for multiple inflammatory CC chemokines. Immunostaining revealed that CCR3 expression was mainly localized to MNV3 lesions of *RNV3 (JR5558)* mice. CCR3^+^ cells included IB4-stained blood endothelium cells, leukocytes, and macrophages but not RPE cells. The expression of CCR3 ligands (CCL11 and CCL24) increased in this mouse model, and they mainly were localized to the subretinal space and the RPE/choroid complex. Intravitreal injection of neutralizing antibodies against CCR3, CCL11, and CCL24 reduced MNV3 area and lesion number in these mice. Systemic administration of the CCR3 antagonists GW766994X and GW782415X had similar effects (Nagai et al., 2015).

## Conclusion and Future Directions

It is already three decades since the discovery of the clinical entity of MNV3 among AMD patients. MNV3 lesions originate from deep retinal vascular plexus, and clinically, it has three stages, including IRN, SRN, and RCA stages. MNV3 can be diagnosed and classified by clinical features, and multimodal imaging includes FA, ICGA, OCT, and OCT-A. Histopathological analysis reveals the expression of VEGF/HIFs, the structure of glomerular IRN, and the existence of intraretinal lipid-filled cells in MNV3 lesions and surrounding cells. These pathological studies suggest the involvement of retinal hypoxia, inflammation, and multiple cell types in the development and progression of MNV3. The structure of glomerular IRN and intraretinal lipid-filled cells are similar to histological features of human retinal hemangioblastoma (RCH), which also has foamy lipid-filled stromal cells.

The mouse models directly related to the hypoxia pathway (retina-specific *VhlKO* mice, *Vegf* overexpression, or *sVEGFR1* knockout in mouse photoreceptor) support the idea that outer retinal hypoxia is an initiation factor for MNV3. The RNV3 (JR5558) model indicates that the involvement of inflammation is necessary but not sufficient for MNV3 development. The *Vldlr^–/–^* mice, *Cyp27a1^–/–^* mice suggest lipid metabolism is vital for MNV3, while the adult EC-specific *SRF* deficiency mouse model supports that aging is a risk factor for MNV3. The finding that Rb can inhibit the Hif activity suggests that smoking or smoking-induced Rb inactivation may play a role in the pathogenesis of MNV3. These animal models also suggest the involvement of photoreceptors, RPE cells, Müller cells, and microglial cells.

Anti-inflammation strategies such as IVTA combined with PDT can improve the visual acuity of MNV3 patients but also induce GA, cataract, and other complications. Other strategies targeting inflammation factors, such as SOCS3, c-Fos, and CCR3/CCL11 or 24, had achieved encouraging results in MNV3 animal models but had not yet been tested in MNV3 patients. Early-stage MNV3 can be treated by anti-VEGF therapy with favorable outcomes, while treatment of late-stage MNV3 is still a challenge. Thus, animal models that can develop retina-originated RCA are important for future studies, which include *Rb/p107/Vhl* TKO mice, RPE-specific *VhlKO* mice, and *Vldlr^–/–^* mice. How subretinal neovascularization broke the RPE and Bruch membrane is the key mechanism, and how to block this process will be the target of future therapy for late-stage MNV3.

A potential candidate target for the future treatment of late-stage MNV3 is the PI3K pathway, which is the downstream signaling pathway of most angiogenesis tyrosine kinase growth factor receptors such as EGFR, VEGFR, and platelet-derived growth factor receptor (PDGFR). PI3K pathway plays crucial roles in cell survival, migration, metabolism, and angiogenesis (Wert et al., 2016; Davies et al., 2019). Blockage of PI3K pathways inhibits retinal neovascularization and improves outcomes (Wert et al., 2016). PI3K pathway can also regulate autophagy to prevent oxidative injury (Zhang et al., 2020) or regulate the Sirtuin 1 (Sirt1)–E2F1 pathway to improve the antioxidative capability of RPE cells (Gong et al., 2020). Thus, it will be interesting to test if PI3K inhibitors can cure MNV3 on these late-stage MNV3 animal models.

## Author Contributions

WQ and DC conceived of and designed the manuscript, and all authors wrote, edited, and approved the manuscript.

## Conflict of Interest

The authors declare that the research was conducted in the absence of any commercial or financial relationships that could be construed as a potential conflict of interest.

## Publisher’s Note

All claims expressed in this article are solely those of the authors and do not necessarily represent those of their affiliated organizations, or those of the publisher, the editors and the reviewers. Any product that may be evaluated in this article, or claim that may be made by its manufacturer, is not guaranteed or endorsed by the publisher.
